# The effect of BCG revaccination on the response to unrelated vaccines in urban Ugandan adolescents (POPVAC C): an open-label, randomised controlled trial

**DOI:** 10.1016/S2214-109X(24)00282-1

**Published:** 2024-10-16

**Authors:** Jacent Nassuuna, Ludoviko Zirimenya, Gyaviira Nkurunungi, Agnes Natukunda, Christopher Zziwa, Caroline Ninsiima, Barbara Apule, Caroline Onen, Susan Amongi, Joel Serubanja, Pius Tumwesige, Denis Nsubuga, Rebecca Amongin, Govert J van Dam, Paul L A M Corstjens, John Kayiwa, Joyce Kabagenyi, Stephen Cose, Anne Wajja, Pontiano Kaleebu, Emily L Webb, Alison M Elliott, POPVAC trial team, Mirriam Akello, Florence A Akello, Hellen Akurut, Susan Amongi, Rebecca Amongin, Barbara Apule, Stephen Cose, Emmanuella Driciru, Alison M Elliott, Joyce Kabagenyi, Joel Kabali, Grace Kabami, Prossy N Kabuubi, Ayoub Kakande, Pontiano Kaleebu, Charity Katushabe, John Kayiwa, Samuel Kiwanuka, Fred Kiwudhu, Robert Kizindo, Moses Kizza, Christine Kukundakwe, Alex Mutebe, Esther Nakazibwe, Loyce Namusobya, Milly Namutebi, Christine Nankabirwa, Beatrice Nassanga, Jacent Nassuuna, Agnes Natukunda, Doreen Nayebare, Caroline Ninsiima, Ronald Nkangi, Gyaviira Nkurunungi, Denis Nsubuga, Ruth Nyanzi, Gloria Oduru, Caroline Onen, Joel Serubanja, Moses Sewankambo, Josephine Tumusiime, Pius Tumwesige, Anne Wajja, Bridgious Walusimbi, Emily L Webb, Ludoviko Zirimenya, Christopher Zziwa

**Affiliations:** a Immunomodulation and Vaccines Focus Area, Vaccine Research Theme, Medical Research Council/Uganda Virus Research Institute and London School of Hygiene & Tropical Medicine Uganda Research Unit, Entebbe, Uganda; b Department of Infection Biology, London School of Hygiene & Tropical Medicine, London, UK; c International Statistics and Epidemiology Group, Department of Infectious Disease Epidemiology, London School of Hygiene & Tropical Medicine, London, UK; d Department of Clinical Research, London School of Hygiene & Tropical Medicine, London, UK; e Department of Parasitology, Leiden University Medical Center, Leiden, Netherlands; f Department of Cell and Chemical Biology, Leiden University Medical Center, Leiden, Netherlands; g Department of Arbovirology, Uganda Virus Research Institute, Entebbe, Uganda; h Department of Global Health and Amsterdam Institute for Global Health and Development, Amsterdam University Medical Centers, Amsterdam, Netherlands

## Abstract

**Background:**

Immune responses induced by several important vaccines differ between populations, with reduced responses in low-income and rural settings compared with high-income and urban settings. BCG immunisation boosts immune responses to some unrelated vaccines in high-income populations. We aimed to test the hypothesis that BCG revaccination can enhance responses to unrelated vaccines in Ugandan schoolchildren.

**Methods:**

We conducted an open-label, randomised controlled trial to compare the effects of BCG revaccination versus no BCG revaccination on the immunogenicity of subsequent unrelated vaccines among adolescents aged 13–17 years who are participants in an urban Ugandan birth cohort study, in which BCG vaccination was documented at birth. Participants were excluded if they had received any of the trial vaccines or related agents when aged 5 years or older. Computer-generated 1:1 randomisation was implemented in REDCap. Participants were excluded if they were concurrently enrolled in other trials; had a clinically significant history of immunodeficiency, or serious psychiatric conditions or moderate to severe acute illnesses; were taking immunosuppressive medications; had allergies to vaccine components, a predisposition towards developing keloid scarring; positive HIV tests or pregnancy tests; were female participants who were lactating; or if they planned to use investigational drugs, vaccines, blood products, or any combination thereof. Trial participants assigned to the BCG revaccination group received the live parenteral BCG-Russia vaccine (Serum Institute of India, Pune, India; 0·1 mL intradermally, right upper arm) at week 0. All participants received yellow fever vaccine (YF-17D; Sanofi Pasteur, Lyon, France; 0·5 mL intramuscularly, left upper arm), live oral typhoid vaccine (Ty21a; PaxVax, London, UK; one capsule per day taken for three alternate days), and quadrivalent virus-like particle human papillomavirus (HPV) vaccine (Merck, Rahway, NJ, USA; 0·5 mL intramuscularly, left upper arm) at week 4; and toxoid vaccines (tetanus–diphtheria; Serum Institute of India; 0·5 mL intramuscularly, left upper arm) and an HPV booster at week 28. An additional HPV vaccination at week 8 was provided to female participants older than 14 years who had not previously been vaccinated. The primary outcomes were yellow fever neutralising antibody titres at 4 weeks post-YF-17D vaccination, *Salmonella enterica* serovar Typhi (henceforth *S* Typhi) O-lipopolysaccharide (O:LPS)-specific IgG concentration at 4 weeks post-Ty21a vaccination, and HPV-16 and HPV-18 L1 protein-specific IgG concentration at 4 weeks post-HPV vaccination. Primary outcome assays were conducted at week 8, and at week 52 for tetanus–diphtheria. We conducted an intention-to-treat analysis comparing log-transformed outcomes between trial groups, with results back-transformed to geometric mean ratios (GMRs). The safety population comprised all randomly allocated participants. The trial was registered at the ISRCTN Registry (ISRCTN10482904) and is complete.

**Findings:**

Between Aug 31 and Oct 12, 2020, we screened 376 potential participants for eligibility. We enrolled and randomly allocated 300 participants to the two groups (151 [50%] to the BCG group and 149 [50%] to the no BCG group). 178 (59%) of 300 participants were male and 122 (41%) were female. 142 (91%) of 151 participants in the BCG group and 139 (93%) of 149 in the no BCG group completed follow-up. There was no effect of BCG revaccination, compared with no BCG revaccination, on the response observed for any vaccine. Yellow fever plaque reduction neutralising reference tests (PRNT_50_) titres (the reciprocal of the last plasma dilution that reduced by 50%) had a GMR of 0·95 (95% CI 0·75–1·19; p=0·62) and PRNT_90_ (reciprocal of the last plasma dilution that reduced by 90%) had a GMR of 0·94 (0·74–1·19; p=0·60); IgG to *S* Typhi O:LPS was 0·99 (0·80–1·23; p=0·94); IgG to HPV-16 was 0·97 (0·69–1·35; p=0·85) and to HPV-18 was 1·03 (0·76–1·40; p=0·83); and toxoid-specific IgG for tetanus was 1·13 (0·87–1·47; p=0·36) and was 1·00 (0·87–1·16; p=0·97) for diphtheria. There were no serious adverse events in either group.

**Interpretation:**

We found no evidence that BCG revaccination is an effective strategy to improve immunogenicity of other vaccines in this low-income, urban setting.

**Funding:**

UK Medical Research Council.

**Translation:**

For the Luganda translation of the abstract see Supplementary Materials section.

**Research in context**

**Evidence before this study**

On April 11, 2024, we searched MEDLINE using the Ovid interface from Jan 1, 1946 to the search date with the following search terms: “(exp BCG vaccine OR exp tuberculosis vaccines) AND (revaccination.mp. or exp Immunization, Secondary/)) AND (vaccin* OR immun* OR antibod* OR humoral OR B-lymphocytes)” in English. A systematic review by Zimmermann and Curtis from 2018 identified eight studies exploring the effect of BCG on humoral responses to 16 unrelated vaccines; results were variable but five studies suggested a positive effect of previous BCG immunisation on the humoral response to other vaccines, including vaccines against hepatitis B, polio type 1, pneumococcus (in neonates in Europe and Africa), and influenza and typhoid (in adults in Russia and the Netherlands). Subsequent trials not included in Zimmerman and Curtis further indicated a beneficial effect on the response to acellular pertussis vaccine in European adult women, but variable effects on a range of SARS-CoV-2 vaccines in Mexico, Brazil, and Peru. A 2023 narrative review by Aaby and colleagues emphasised the possibility of enhanced non-specific BCG effects with multiple BCG doses, and of variability of effects between BCG strains and the broader environment.

**Added value of this study**

In the Population Differences in Vaccine Responses (POPVAC) trial C, we conducted an open-label, randomised, controlled trial of the effect of BCG revaccination among Ugandan adolescents who received BCG at birth on the humoral response to five vaccines (live and inert, and parenteral and oral) that are relevant to this age group in Africa: yellow fever (YF-17D), oral typhoid (Ty21a), and human papillomavirus, all given 4 weeks after BCG; and a tetanus–diphtheria booster given 24 weeks after BCG. We found no effect of BCG revaccination on response to any of these vaccines.

**Implications of all the available evidence**

BCG vaccination or revaccination might have beneficial effects on the humoral response to unrelated vaccines, but these effects are variable. Factors that determine benefit could include the following: age group; broader environmental exposures of people who are to be vaccinated; BCG strain and dose number; and the characteristics, timing, combinations, and sequence of subsequent vaccines. However, our POPVAC C trial results, and overall findings of the POPVAC programme, suggest that BCG revaccination is unlikely to be an effective strategy to address the challenge of reduced vaccine responses in low-income countries.

## Introduction

Vaccines are a key tool to protect against infectious diseases, but there is increasing evidence that the immunogenicity and efficacy of important licensed and candidate vaccines vary between populations, with lower responses characteristic of low-income, tropical, and particularly in rural settings.^1^

We established the Population Differences in Vaccine Responses (POPVAC) programme of three randomised controlled trials to investigate variation in vaccine responses in settings in urban and rural Uganda,^2^ directing our attention in particular to the effects of chronic, immunomodulating parasitic infections. In the POPVAC A^3^ and POPVAC B^4^ trials, we investigated whether the modulation of vaccine responses by current schistosomiasis and malaria parasitic infections could be reversed by intensive treatment. However, while animal models and observational studies in humans have provided evidence that these parasites have an effect on unrelated vaccine responses, interventional studies, including our trials,^3^, ^4^ have shown surprisingly small or transient reversal of such effects.^5^, ^6^ This suggests that both past and active parasitic co-infections, and a broader spectrum of repeated microbial or other environmental exposures, can shape the immune system to determine population vaccine responses in ways that cannot easily be reversed.^1^ Live vaccines can be considered model infections and can be used experimentally to provide insights into the non-specific ways in which infections affect the immune system; they can be used to explore how an infection exposure influences the response to unrelated vaccines.

Evidence that has accumulated over several decades suggests that live vaccines, including BCG, measles, and *Vaccinia*, can have substantial, enduring, non-specific, and largely beneficial health effects.^7^, ^8^ These could be mediated through modulation of the innate immune system: studies, particularly on BCG, show evidence of lasting epigenetic modification of monocytes, macrophages, and natural killer cells. These modifications are associated with enhanced responses to unrelated antigens, a process termed trained innate immunity. Benefits might include enhanced innate control of unrelated pathogens, improved induction of antibodies, and down-modulation of excessive inflammatory responses.^9^ Additionally, effects on the adaptive immune system—such as heterologous immunity, in which T cells are activated in an antigen-independent manner by circulating cytokines,^9^ or molecular mimicry, in which vaccine-induced, adaptive responses also recognise antigens from organisms that are unrelated but have a similar structure^10^—could provide non-specific vaccine-induced protection against unrelated infections.

The effects of combining vaccines are not always positive and the occurrence of interference is well recognised, in which live vaccines, given together or within a short time period, can sometimes reduce the responses induced by each other. For this reason, WHO recommends that live vaccines (such as measles and yellow fever vaccine) be given at least 4 weeks apart.^11^ However, a systematic review published in 2018 described evidence that live BCG vaccine can enhance responses to several other unrelated vaccines.^12^ Results varied between populations and vaccine types; for example, previous vaccination with BCG enhanced responses to some vaccines given in infancy in some trials, and studies among European and Russian adults showed enhanced responses to inactivated influenza vaccine.^12^ A more recent study among adult Dutch women showed enhanced responses to pertussis vaccine.^13^ Studies also suggest that exposure to multiple different live vaccines or multiple doses of the same vaccine (eg, BCG revaccination following BCG at birth) might produce stronger non-specific effects than a single dose of a live vaccine.^7^

Prevention of tuberculosis was the original target of BCG vaccination, and interest in BCG revaccination for protection against pulmonary tuberculosis in adolescents and young adults has been rekindled by recent studies suggesting protection against *Mycobacterium tuberculosis* infection^14^ as well as disease.^15^ Thus, a combination of *M tuberculosis-*specific and non-specific benefits could make BCG revaccination a valuable component of the vaccine portfolio for schoolchildren in countries with a high burden of infection.

In POPVAC C, we aimed to test the hypothesis that BCG revaccination improves responses to unrelated vaccines among schoolchildren in a low-income African setting.^16^ We enrolled participants from the Entebbe Mother and Baby Study birth cohort (EMaBS),^17^ among whom BCG vaccination, including details of BCG strain, was documented at birth, and we undertook a nested trial of BCG revaccination versus no BCG revaccination in adolescence. The intervention was followed by a portfolio of vaccines selected as representative of live and inert, parenteral, and oral vaccines that are of value among African school-age children to determine whether BCG revaccination enhances responses to unrelated vaccines in this setting.

## Methods

### Study design and participants

The POPVAC C trial protocol has been published previously.^16^ We conducted an open-label, randomised controlled trial in Entebbe municipality, Wakiso district, Uganda, a predominantly urban setting. The trial recruited participants from the EMaBS birth cohort, comprising children born to 2507 pregnant women enrolled in their second or third trimester between 2003 and 2005 for a trial of anthelmintic treatment during pregnancy and early childhood. It aimed to investigate the effects on childhood vaccine responses and infectious disease incidence. Between Aug 31 and Oct 12, 2020, we recruited participants aged 13–17 years from EMaBS.

Participants were eligible if they agreed to remain within the study area and contactable throughout the trial, and to adhere to all study requirements. For female participants, they also needed to agree to avoid pregnancy during the trial. At birth, the child's sex was recorded in the EMaBS cohort. At enrolment into POPVAC C, we documented sex again using self-report. Participants were excluded if they were concurrently enrolled in other trials; had a clinically significant history of immunodeficiency, serious psychiatric conditions, or moderate or severe acute illnesses; had been vaccinated with any of the trial vaccines or related agents when aged 5 years or older; were taking immunosuppressive medications; had allergies to vaccine components; were susceptible to keloid scarring; had positive HIV tests or pregnancy tests; were female participants who were lactating; or planned to use investigational drugs, vaccines, blood products, or any combination thereof to the end of follow-up.

All participants and their parents or guardians gave written informed assent and consent, respectively. Ethics approval was granted by the Uganda Virus Research Institute (reference GC/127/18/09/682), the London School of Hygiene & Tropical Medicine (reference 16034), the Uganda National Council for Science and Technology (reference HS2491), and the Uganda National Drug Authority (reference CTA0094). The independent Programme Steering Committee and Data and Safety Monitoring Board oversaw the trial. The trial was registered at the ISRCTN Registry (ISRCTN10482904).

### Randomisation and masking

An independent statistician used randomly permuted blocks (sizes 4, 6, 8, and 10) to generate a randomisation code used to assign participants in a 1:1 ratio to receive either the live parenteral BCG vaccine or no BCG vaccine. This code was embedded into an electronic data capture system (REDCap version 13.1.08 Vanderbilt University, Nashville, TN, USA) which was used to allocate the codes sequentially to eligible participants at enrolment, and the participant received either BCG vaccine or not according to this allocation. The immunology laboratory staff assessing the trial outcomes were masked to treatment allocation. As an open-label trial, participants and clinicians were not masked to treatment.

### Procedures

**Figure 1:**
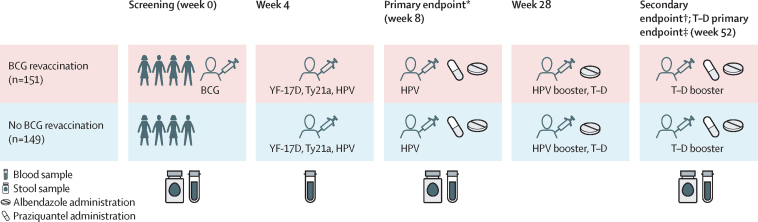
Trial schedule Samples were collected before vaccinations or anthelmintic treatment at relevant timepoints. YF-17D=yellow fever vaccine. Ty21a=live oral typhoid vaccine. HPV=virus-like particle human papillomavirus vaccine. T–D=tetanus–diphtheria vaccine. Created with BioRender.com. *Primary endpoint following BCG, YF-17D, Ty21a, and HPV vaccination; additionally, an HPV dose was given to previously unvaccinated girls aged 14 years or older. †Secondary endpoint following BCG, YF-17D, Ty21a, and HPV vaccination. ‡A T–D boost was given to comply with the Uganda National Expanded Program on Immunization guidelines.

All clinical assessments and vaccinations were performed at the EMaBS clinic, an extension of the Entebbe General Hospital. Trial participants assigned to the BCG revaccination group were vaccinated with the live parenteral BCG-Russia vaccine (Serum Institute of India Pune, India; 0·1 mL intradermally, right upper arm) at week 0. All participants received yellow fever vaccine (YF-17D; Sanofi Pasteur, Lyon, France; 0·5 mL intramuscularly, left upper arm); live oral typhoid vaccine (Ty21a; PaxVax, London, UK; one capsule per day taken for three alternate days), and quadrivalent virus-like particle human papillomavirus (HPV) vaccine (Merck, Rahway, NJ, USA; 0·5 mL intramuscularly, left upper arm) at week 4; and toxoid vaccines (tetanus–diphtheria; Serum Institute of India; 0·5 mL intramuscularly, left upper arm) and an HPV booster at week 28 (figure 1). To comply with prevailing government guidelines, an additional HPV vaccination at week 8 was provided to female participants aged 14 years or older who had not previously been vaccinated, after study samples had been collected.

At screening, sociodemographic and clinical variables were collected, and blood samples were obtained for malaria testing (a positive PCR for *Plasmodium falciparum* infection; assay details provided in appendix 2 pp 15–16), modified Knots testing for *Mansonella perstans*, full blood count, HIV serology, testing for previous malarial infection and *Schistosoma mansoni* infection, and assessment of pre-vaccination parameters. Blood samples were collected at screening and at weeks 4, 8, 28, and 52 for measurement of vaccine responses and exploratory immunology. Stool samples were obtained at screening and at weeks 8 and 52 to determine helminthic infections (figure 1). *S mansoni* infection status was determined retrospectively through plasma measurement of circulating anodic antigen (CAA) and stool *S mansoni* DNA detection with a multiplex PCR assay that also included primers and probes for *Necator americanus* and *Strongyloides stercoralis* DNA detection (appendix 2 pp 8–10).

Albendazole and praziquantel were provided to all participants at weeks 8 and 52, as per Ugandan national guidelines to manage nematode and trematode infections, respectively (figure 1).

### Outcomes

The primary outcomes were yellow fever neutralising antibody titres at 4 weeks post-YF-17D vaccination, *Salmonella enterica* serovar Typhi (henceforth *S* Typhi) O-lipopolysaccharide (O:LPS)-specific IgG concentration at 4 weeks post-Ty21a vaccination, and HPV-16-specific and HPV-18-L1 protein-specific IgG concentration at 4 weeks post-HPV vaccination. Primary outcome assays were conducted at week 8. Our original protocol specified assessment of baseline levels of tetanus and diphtheria toxoid-specific IgG concentrations at week 28, before tetanus–diphtheria administration, and primary outcome assay for tetanus–diphtheria at week 32, 4 weeks after the immunisation. However, this schedule was amended based on programmatic and protocol changes during the COVID-19 pandemic, and also for financial considerations. Thus, the primary outcome for tetanus–diphtheria was assessed 24 weeks after vaccination, at week 52 of the trial.

Plasma neutralising antibodies against yellow fever virus (obtained from WHO Yellow Fever Regional Reference Laboratory, Institut Pasteur, Dakar, Senegal) were assessed using plaque reduction neutralising reference tests (PRNTs, appendix 2 p 12). We report PRNT_50_ and PRNT_90_ titres, defined as the reciprocal of the last plasma dilution that reduced by 50% or 90%, respectively, the number of virus plaques infected by 100 plaque-forming units per 0·1 mL of the reference 17D virus preparation. For assay quality control, 50% and 90% neutralisation cutoff values were determined in each assay by back titration of the virus inoculum. Plasma HPV-16-specific and HPV-18-specific IgG antibodies were measured by ELISA, using virus-like particles from Frederick National Laboratory for Cancer Research (Frederick, MD, USA). *S* Typhi O:LPS-specific IgG levels were quantified by ELISA, using standards from the Oxford Vaccine Centre Biobank (Oxford, UK). To constitute the standards, we used a pooled sample generated from sera of known O:LPS-specific IgG titres. These sera had been collected from the highest responders to O-antigen following the challenge with *S* Typhi in a controlled human infection study.^18^ Anti-diphtheria and anti-tetanus IgG levels were also determined by ELISA, using WHO reference preparations and standards. Detailed methods are given in appendix 2 (pp 13–15).

Planned secondary outcomes were as follows: assessment of response waning by measurement of the primary outcomes described above but at week 52 (for all vaccinations except tetanus and diphtheria) and area under the curve (AUC) combining week 8 and week 52 responses; the proportion of participants with protective neutralising antibodies for yellow fever, protective IgG levels for tetanus toxoid, and seroconversion for Ty21a at 4 weeks after immunisation (24 weeks for tetanus toxoid); and effects of the intervention on priming versus boosting for HPV only, comparing outcomes at 4 weeks after the first dose with outcomes at week 52. Participants with PRNT_50_ titres of 10 or greater following YF-17D vaccination were considered seropositive.^19^ Tetanus toxoid-specific IgG levels of 0·1 international units (IUs) per mL or greater after tetanus–diphtheria vaccination^20^ were considered protective. Seroconversion following Ty21a vaccination was defined as a 4-fold or greater increase in *S* Typhi O:LPS-specific IgG over baseline.^21^

In addition, we assessed BCG-specific interferon-γ (IFNγ) responses (our intervention) using freshly isolated peripheral blood mononuclear cells (PBMCs) and a Human IFNγ (ALP) ELISpot Flex kit (Mabtech, Stockholm, Sweden). Assay details are documented in appendix 2 (pp 10–11). We report results as spot-forming units (SFUs) per million PBMCs, calculated sequentially by subtracting mean SFUs of unstimulated assay wells from mean SFUs of duplicate BCG-stimulated wells, and then by correcting for the number of PBMCs (300 000) per well. Samples that had more than 83·3 SFUs per million PBMCs in either of the duplicate unstimulated well were considered invalid and not included in the final analysis. We performed quality control on all ELISpot plate samples, in which we checked that the spot counts for the negative and positive control wells were within the acceptable set range, among other parameters (appendix 2 pp 8–16). A small subset of participants in the no BCG group were included for ELISpot assays at each timepoint in order not to unmask the laboratory team to trial group. The safety population comprised all randomly allocated participants.

### Statistical analysis

We hypothesised that standard deviations of primary outcome measures would lie between 0·3 log_10_ and 0·6 log_10_,^22^ and that revaccination with BCG might increase responses by approximately 0·12 log_10_ to 0·14 log_10_.^23^ The target sample size of 300 participants (150 per group) was determined to give over 80% power to detect differences of 0·12 log_10_ units (SD 0·3) to 0·22 log_10_ units (SD 0·6) in vaccine response at a 5% significance level, allowing for 10% loss to follow-up.

Baseline characteristics of participants and proportions receiving each vaccination were summarised by trial group. Analysis was done by intention to treat so that participants were analysed in their randomised group regardless of whether they received BCG or not. The analysis population for each vaccine-specific outcome included all participants who received the vaccine corresponding to that outcome. For Ty21a, we included participants who received at least one of the three doses. For the secondary outcome of priming versus boosting of HPV, we included participants who received both HPV doses at weeks 4 and 28 and no additional doses.

Primary outcomes were log_10_ transformed and compared between trial groups using unpaired Student's *t* tests, with results back-transformed to give geometric mean ratios (GMRs) and 95% CIs. For the secondary outcomes of response waning of YF-17D, Ty21a, and HPV, we compared responses at week 52, and AUC from week 8 and week 52 responses, using the same approach as described for the primary outcomes. Protective immunity outcomes for YF-17D, Ty21a, and tetanus were summarised as proportions and compared between trial groups as differences in proportions and corresponding 95% CIs. The effect of BCG revaccination on priming versus boosting for HPV was assessed by including an interaction between timepoint and trial group in a mixed effects linear regression model with HPV antibody at week 8 and week 52 as the outcome. No adjustment for covariates was made in the primary analyses, but in exploratory analyses we investigated the effect of controlling for the corresponding vaccine response at baseline. In a planned subgroup analysis, we assessed whether the effect of BCG revaccination on primary outcomes differed by sex, using linear regression of log-transformed outcomes and including an interaction term between trial group and participant sex. Analyses and data visualisation were done in Stata version 17 and GraphPad version 9.0.0.

### Role of the funding source

The funder of the study had no role in study design, data collection, data analysis, data interpretation, or writing of the report.

## Results

**Figure 2:**
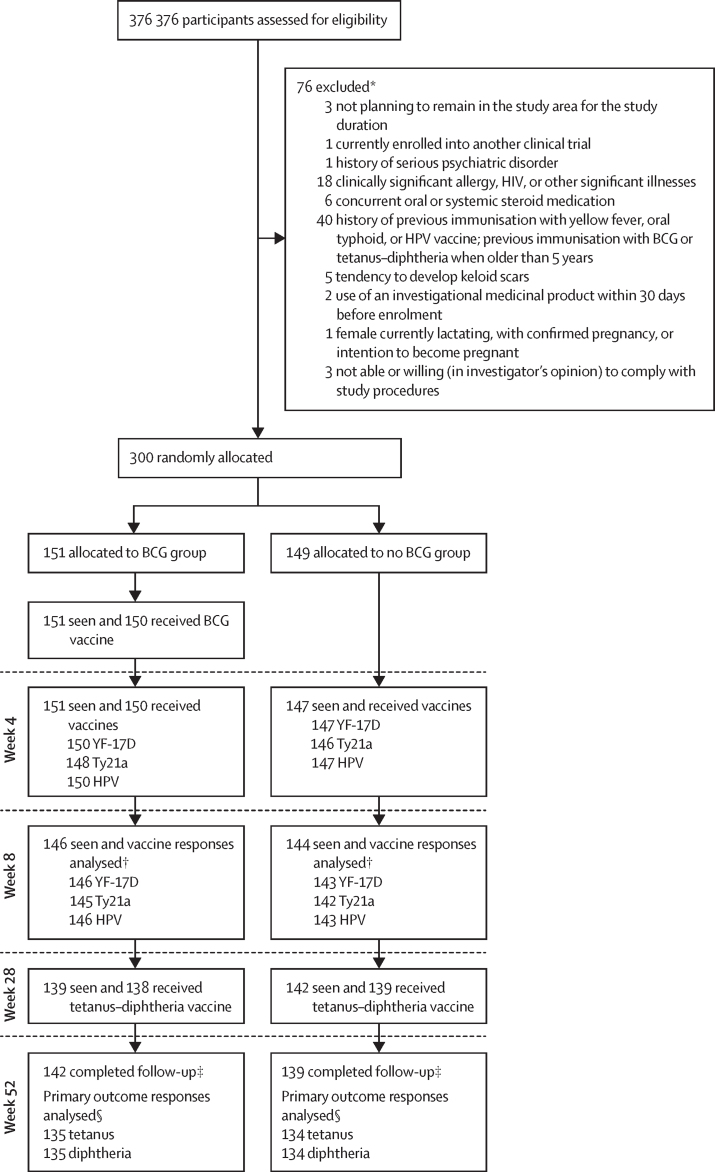
Trial profile YF-17D=yellow fever vaccine. Ty21a=live oral typhoid vaccine. HPV=virus-like particle human papillomavirus vaccine. *Some participants had multiple reasons for exclusion. †In the BCG group, one participant did not receive Ty21a vaccine. In the no BCG group, one participant did not receive the Ty21a vaccine and one participant had no blood sample. ‡Nine participants did not complete follow-up in the BCG group, and ten participants did not complete follow-up in the no BCG group, for a total of 19 participants. The reasons for withdrawal were loss to follow-up (n=14), development of a medical condition that prohibited their continuing in the trial (n=4), and withdrawal of consent (n=1). §In the BCG group, six participants did not receive the tetanus–diphtheria vaccine and one participant had no blood sample. In the no BCG group, five participants did not receive the tetanus–diphtheria vaccine.

Between Aug 31 and Oct 12, 2020, we screened 376 potential participants for eligibility. We enrolled and randomly allocated 300 participants to the two groups (151 [50%] to the BCG group and 149 [50%] to the no BCG group). The trial profile is shown in figure 2.

The baseline characteristics of the trial participants are shown in table 1. The participants had a median age of 15 years (IQR 15–16). 178 (59%) of 300 participants were male and 122 (41%) were female. In the BCG group, 26 (17%) of 151 and 48 (32%) of 148 participants had *S mansoni* infection by CAA and PCR, respectively, with three participants missing samples that could undergo PCR; and 12 (8%) of 151 participants had malaria infection at baseline, detected by PCR for *Plasmodium falciparum*. In the no BCG group, 32 (21%) of 149 and 59 (40%) of 149 participants had *S mansoni* infection by CAA and PCR, respectively; six (4%) of 149 participants had malaria infection at baseline, detected by PCR for *P falciparum*. Participants in the BCG group had lower BCG-specific IFNγ SFUs per 1 million PBMCs at baseline than those in the no BCG group (46·7 *vs* 60·8). A smaller number of participants in both groups were included in analyses of HPV, as female participants who had already received HPV at school did not receive the vaccine again during the study. Further participant characteristics are shown in appendix 2 (p 5).

**Table 1:** Baseline demographic and clinical characteristics and vaccine responses

		**BCG group (n=151)**	**No BCG group (n=149)**
Age in years, median (IQR)		15 (15 to 16)	15 (15 to 16)
Sex			
	Male	88 (58%)	90 (60%)
	Female	63 (42%)	59 (40%)
BMI in kg/m^2^, median (IQR)		20·2 (18·6 to 22·0)	19·8 (18·3 to 22·2)
BCG scar present		97 (64%)	80 (54%)
BCG strain received at birth*			
	Russia	65/137 (47%)	66/135 (49%)
	Bulgaria	61/137 (45%)	59/135 (44%)
	Denmark	11/137 (8%)	10/135 (7%)
Helminth infections at baseline			
	*Schistosoma mansoni*, CAA ≥30 pg/mL	26/151 (17%)	32/149 (21%)
	*Schistosoma mansoni*, PCR positive	48/148† (32%)	59/149 (40%)
	*Necator americanus*, PCR positive	1/148† (1%)	2/149 (1%)
	*Strongyloides stercoralis*, PCR positive	0/148†	2/149 (1%)
Malaria infection at baseline			
	*Plasmodium falciparum*, PCR positive	12 (8%)	6 (4%)
Sensitisation to *Mycobacterium tuberculosis*‡			
	ESAT-6-specific and CFP-10-specific IFNγ in SFUs per 1 million PBMCs, median (IQR)	31·7 (11·7 to 68·3)	36·7 (13·3 to 81·7)
Baseline vaccine responses, median (IQR)			
	BCG-specific IFNγ, SFUs per 1 million PBMCs§	46·7 (26·7 to 83·3)	60·8 (35·0 to 131·7)
	Yellow fever PRNT_50_ titres	<10 (<10 to <10)	<10 (<10 to <10)
	*S* Typhi O:LPS-specific IgG, EU/mL	232·3 (93·4 to 425·9)	201·9 (101·8 to 350·4)
	HPV-16-specific IgG, EU/mL	3·0 (2·2 to 5·2)	3·2 (2·0 to 4·6)
	HPV-18 specific IgG, EU/mL	60·2 (43·4 to 91·2)	62·2 (40·2 to 101·4)
	Tetanus toxoid-specific IgG, IU/mL	0·16 (0·07 to 0·71)	0·149 (0·07 to 0·56)
	Diphtheria toxoid-specific IgG, IU/mL	0·07 (0·02 to 0·23)	0·060 (0·01 to 0·22)

Data are n (%) unless otherwise stated. CFP-10=10-kDa culture filtrate protein. ESAT-6=6-kDa early secretory antigenic target. EU=ELISA unit. HPV=human papillomavirus. IU=international unit. O:LPS=O-lipopolysaccharide. PMBC=peripheral blood mononuclear cells. PRNT_50_=plaque reduction neutralising reference tests, for the reciprocal of the last plasma dilution that reduced by 50%. SFU=spot-forming units. *S* Typhi=*Salmonella enterica* serovar Typhi.

* Information not available for 28 children delivered in hospitals other than Entebbe hospital.

† Three participants missing PCR sample data.

‡ A 1:1 mix of the ESAT-6 and CFP-10 was used for exploratory assessment of tuberculosis infection.

§ Baseline vaccine responses not available for 17 participants for BCG-specific IFNγ (ten participants in the BCG group and seven participants in the no BCG group) and ten participants for tetanus–diphtheria at week 8 (five participants in the BCG group and five participants in the no BCG group).

Of the 151 participants allocated to the BCG group, 150 (99%) received the BCG vaccine at week 0. 151 (100%) participants received YF-17D, 148 (98%) received Ty21a, and 150 (99%) received the HPV vaccine at week 4; 138 (91%) received the tetanus–diphtheria vaccine at week 28; and 142 (91%) participants completed follow-up. Of the 149 participants enrolled in the no BCG group, 147 (99%) received YF-17D and HPV vaccines, and 146 (98%) received Ty21D; 139 (93%) received the tetanus–diphtheria vaccine at week 28; and 139 (93%) completed follow-up (figure 2). The proportion of participants who received vaccines across the different timepoints was similar between trial groups (appendix 2 p 5).

We observed no effect of BCG revaccination on antibody responses for any of the study vaccines at the primary outcome timepoints (table 2). Yellow fever PRNT_50_ titres had a GMR of 0·95 (95% CI 0·75–1·19; p=0·62); PRNT_90_ titres had the same GMR (95% CI 0·74–1·19; p=0·60); IgG to *S* Typhi O:LPS was 0·99 (0·80–1·23; p=0·94); IgG to HPV-16 was 0·97 (0·69–1·35; p=0·85) and to HPV-18 was 1·03 (0·76–1·40; p=0·83); and toxoid-specific IgG for tetanus was 1·13 (0·87–1·47; p=0·36) and for diphtheria was 1·00 (0·87–1·16; p=0·97; appendix 2 p 4). In exploratory analyses, adjusting for baseline differences in BCG-specific IFNγ ELISpot responses had no effect.

**Table 2:** Effect of BCG revaccination versus no BCG revaccination in Ugandan adolescents, primary endpoint analysis

		**n**	**Geometric mean (SE)**	**Geometric mean ratio (95% CI)**	**p value**
**Primary endpoint analysis**					
Yellow fever PRNT_50_ titres (8 weeks post-vaccination)					
	BCG	146	3082·76 (1·09)	0·94 (0·75–1·19)	0·62
	No BCG	143	3263·22 (1·08)	ref	..
Yellow fever PRNT_90_ titres (8 weeks post-vaccination)					
	BCG	146	293·72 (1·09)	0·94 (0·74–1·19)	0·60
	No BCG	143	312·38 (1·08)	ref	..
*S* Typhi O:LPS-specific IgG (8 weeks post-vaccination), EU/mL					
	BCG	145	835·98 (1·08)	0·99 (0·80–1·23)	0·94
	No BCG	142	843·34 (1·08)	ref	..
HPV-16-specific IgG (8 weeks post-vaccination), EU/mL					
	BCG	146	108·84 (1·13)	0·97 (0·69–1·35)	0·85
	No BCG	143	112·50 (1·12)	ref	..
HPV-18-specific IgG (8 weeks post-vaccination), EU/mL					
	BCG	146	556·47 (1·11)	1·03 (0·76–1·40)	0·83
	No BCG	143	537·98 (1·12)	ref	..
Tetanus toxoid-specific IgG (52 weeks post-vaccination), IU/mL					
	BCG	135	13·45 (1·10)	1·13 (0·87–1·47)	0·36
	No BCG	134	11·91 (1·10)	ref	..
Diphtheria toxoid-specific IgG (52 weeks post-vaccination), IU/mL					
	BCG	135	2·01 (1·05)	1·00 (0·87–1·16)	0·97
	No BCG	134	2·00 (1·05)	ref	..
**Secondary endpoint analysis**					
Yellow fever PRNT_50_ titres (52 weeks post-vaccination), EU/mL					
	BCG	143	2151·75 (1·09)	1·13 (0·88–1·44)	0·34
	No BCG	139	1909·33 (1·10)	ref	..
Yellow fever PRNT_90_ titres (52 weeks post-vaccination), EU/mL					
	BCG	143	187·50 (1·09)	1·11 (0·88–1·41)	0·37
	No BCG	139	168·19 (1·09)	ref	..
*S* Typhi O:LPS-specific IgG (52 weeks post-vaccination), EU/mL					
	BCG	141	236·51 (1·09)	0·97 (0·77–1·22)	0·80
	No BCG	138	243·60 (1·08)	ref	..
HPV-16-specific IgG (52 weeks post-vaccination), EU/mL					
	BCG	86	431·58 (1·12)	1·01 (0·73–1·41)	0·94
	No BCG	92	425·92 (1·13)	ref	..
HPV-18-specific IgG (52 weeks post-vaccination), EU/mL					
	BCG	86	847·90 (1·10)	0·92 (0·70–1·22)	0·57
	No BCG	92	918·88 (1·11)	ref	..

EU=ELISA unit. HPV=human papillomavirus. IU=international unit. O:LPS=O-lipopolysaccharide. PMBC=peripheral blood mononuclear cells. PRNT_50_=plaque reduction neutralising reference tests, for the reciprocal of the last plasma dilution that reduced by 50%. PRNT_90_=plaque reduction neutralising reference tests, for the reciprocal of the last plasma dilution that reduced by 90%. *S* Typhi=*Salmonella enterica* serovar Typhi.

Secondary outcome analyses also showed no effect of BCG revaccination on any of the study vaccine responses at week 52 (table 2): yellow fever neutralising antibody (GMR 1·13 [95% CI 0·88–1·44]; p=0·34); *S* Typhi O:LPS-specific IgG (0·97 [0·77–1·22]; p=0·80); and IgG to HPV-16 (1·01 [0·73–1·41]; p=0·94) and to HPV-18 (0·92 [0·70–1·22]; p=0·57; appendix 2 p 4). There was no effect of BCG revaccination on AUC based on week 8 and week 52 responses, and no effect on the proportions with protective response levels for yellow fever, Ty21a, or tetanus (appendix 2 p 6). No serious or severe adverse events were observed (table 3). Planned subgroup analyses showed no differences in the effect of BCG revaccination when stratified by sex (appendix 2 p 7). Administration of the BCG vaccine increased the BCG-specific IFNγ responses in individuals in the BCG group across the different timepoints (geometric mean 48·3 [95% CI 41·3–56·4] at week 0 *vs* 193·9 [95% CI 171·4–219·6] at week 8 [p=0·001] *vs* 91·6 [95% CI 78·7–106·5] at week 52 [p<0·0001 compared with week 0]; appendix 2 p 3).

**Table 3:** Summary of the adverse events

	**BCG group (n=151)**		**No BCG group (n=149)**	
	All adverse events	Adverse event possibly, probably, or definitely related to intervention*	All adverse events	Adverse event possibly, probably, or definitely related to intervention*
Participants with at least one adverse event	9 (6%)	9 (6%)	4 (3%)	4 (3%)
Total number of adverse events, n (number of participants with a grade 3 event)	12 (0)	12 (0)	5 (0)	5 (0)
Average number of adverse events per participant with adverse events†	1·3	1·3	1·3	1·3
Participants with a serious adverse event	0	0	0	0

Data are n (%) unless otherwise stated.

* Relation to any study intervention in the opinion of study investigators.

† Calculated as the number of adverse events divided by the number of subjects experiencing any adverse event.

## Discussion

In the POPVAC C trial, there was no effect of BCG revaccination on the humoral response to subsequent live vaccines (yellow fever or oral typhoid) or inert vaccines (HPV, tetanus, or diphtheria). There was no evidence of a difference in the effect of BCG revaccination by sex.

By chance, the BCG ELISpot response in the no BCG group was slightly higher at baseline than in the BCG revaccination group, but adjusting for this had no effect on our analyses, so it is unlikely that this baseline difference was responsible for any absence of effect of the intervention.

Previous studies on the effects of BCG vaccination or revaccination on unrelated vaccine responses have shown variable results.^12^ BCG vaccination in infancy showed evidence of enhanced responses to polio and hepatitis B vaccines in The Gambia,^12^ and higher responses to pneumococcal vaccines but lower responses to hepatitis B vaccines in Australia,^24^ and no effect on a range of vaccines studied in Denmark and Senegal.^12^ Among adults, besides evidence of positive effects on influenza and pertussis vaccine responses in Europe,^13^, ^25^ BCG vaccination or revaccination enhanced subsequent responses to the SARS-CoV-2 COVISHIELD vaccine in India^26^ and Pfizer–BioNTech in Mexico,^27^ but not to the SARS-CoV-2 Oxford–AstraZeneca or Sinovac vaccines in a large study in Brazil.^28^ There is also no compelling evidence that BCG vaccination or revaccination reduces COVID-19 disease incidence or severity.^29^, ^30^ Several factors could have contributed to this variability, and to the absence of effect observed in our study.

First, the BCG strain used might have had an effect. Most previous studies in adults have used the Danish BCG strain. In POPVAC C, we enrolled participants who had received either BCG-Denmark, BCG-Bulgaria, or BCG-Russia at birth; the latter was the most widely used strain in Uganda at the time but, in cohort participants, was associated with lower *M tuberculosis*-specific responses and less scarring, as well as lower responses to tetanus toxoid than was seen among recipients who, by chance, received Danish BCG.^31^ Although all participants in POPVAC C had been vaccinated at birth, only 64% of participants had BCG scars at enrolment to the trial in adolescence. In our study, the use of BCG-Russia in adolescence, and in some participants at birth, might have attenuated the expected BCG effects, although BCG-Russia showed a positive effect on the antibody response to COVISHIELD in India.^26^

Second, effects might have been masked or blocked by other, cumulative environmental exposures. This been much debated in relation to the tuberculosis-specific effects of BCG, and it is notable that the efficacy of BCG in infancy against tuberculosis appears less affected by environmental differences than its efficacy at school age, suggesting that masking or blocking is acquired with age.^15^ Early studies on the non-specific benefits of BCG focused on infancy and early childhood, so non-specific effects might similarly decline with age in low-income country settings, where microbial—including mycobacterial—exposure is intense.^32^ The European studies showing BCG-induced enhancement of influenza and pertussis responses in adults are probably at less risk of such potential masking effects. Baseline BCG-specific sensitisation was high among EMaBS participants, indicating the potential for masking or blocking of revaccination effects (preventing or obscuring protection against TB specifically, or non-specific enhancement of vaccine responses or protection against other organisms).

Third, events occurring after the BCG revaccination, including within the trial, might have interfered with the possibility of observing non-specific effects. 4 weeks post-BCG, participants received yellow fever and oral Ty21a typhoid vaccines (both live) and HPV (a viral particle vaccine) together on the same day, with additional Ty21a doses 2 and 4 days later. Interference between vaccines can occur when they are given on the same day,^33^ and could have overridden the effects of BCG on the three vaccines given together; subsequent vaccinations, whether live or inert, might override the non-specific effects of previous BCG.^7^ Thus, non-specific effects of vaccination with yellow fever, Ty21a, and HPV at week 4 could have prevented any effect of BCG on the response to tetanus or diphtheria booster vaccinations at week 28.

Our trial had some limitations. It was conducted within an urban birth cohort that has lost some cohort members to follow-up since birth. The results might not be generalisable to rural settings, and our participants might not fully represent the entire Entebbe area. Additionally, while the subgroup analyses were pre-specified, our study might not have had sufficient power to detect large differences between subgroups, hence it is possible that we did not detect genuine differences in the effect of BCG revaccination by sex. Only one participant allocated to BCG did not receive the vaccine; therefore we provide only the intention-to-treat analysis. The difference in an additional per-protocol analysis would be negligible.

Strengths of our trial include the randomised trial design, substantial sample size, and less than 10% loss to follow-up. Nesting of the work in the EMaBS cohort introduced bias resulting from differential loss to follow-up during the years since birth, but allowed certainty regarding previous vaccinations and the BCG strain used. Inclusion of a portfolio of vaccines post-intervention allowed us to explore potential effects on different types of vaccine, but precluded the possibility of examining effects of BCG alone on other individual vaccines. Although children in Entebbe are exposed from early life to a broader range of infections than their European counterparts, their exposure is less intense than in rural Uganda.^34^ Thus, further studies might be considered to test the effect of BCG revaccination alone on the response to an individual vaccine of particular interest in specific populations who are at high risk.

Taken together, our results indicate that BCG revaccination given 4 weeks before a portfolio of unrelated vaccines of relevance to school-age children in Uganda does not result in interference, but we also found no evidence that BCG revaccination enhanced antibody responses to any of the vaccines in our portfolio. Moreover, across the POPVAC programme, responses to several other vaccines were lower in the more rural schistosomiasis-endemic and malaria-endemic settings than in the more urban Entebbe setting,^34^ despite inclusion of BCG vaccination or revaccination 4 weeks before the other vaccines. Our findings do not suggest that BCG would be a useful solution to the challenge of impaired vaccine immunogenicity in at-risk populations in low-income tropical settings.

### POPVAC trial team

### Contributors

### Equitable partnership declaration

### Data sharing

## Declaration of interests

GN reports funding from the Wellcome Trust and from the EDCTP2 programme supported by the EU. AME, SC, and PK report funding from the UK Medical Research Council (MRC) for conduct of the study; AME and SC report funding from DELTAS Africa, outside the submitted work, and support from the UK National Institute for Health and Care Research (NIHR). AME reports funding from the Science for Africa Foundation, outside the submitted work. AME and SC further report support from the Serum Institute of India, Uganda National Expanded Program on Immunization, and Emergent BioSolutions for conduct of the study. All other authors declare no competing interests.

## Supplementary Materials

Luganda translation of the abstract

Supplementary appendix 2

Equitable Partnership Declaration
