# Supplementary material for: The effect of BCG revaccination on the response to unrelated vaccines in urban Ugandan adolescents (POPVAC C): an open-label, randomised controlled trial
Source: Lancet Glob Health. 2024 Oct 16;12(11):e1849–59. doi: 10.1016/S2214-109X(24)00282-1 (PMC11483248; doi:10.1016/S2214-109X(24)00282-1)
Supplement: Luganda translation of the abstract [file mmc1.pdf]

THE LANCET
Global Health

Supplementary appendix 1

This translation in Luganda was submitted by the authors and we reproduce it as supplied. It has not been peer reviewed. The Lancet's editorial processes have only been applied to the original in English, which should serve as reference for this manuscript.

Ekiwandiiko kino ekikyuse mu lulimi Oluganda kyaweereddwayo abawandiisi b'ennyini era tukifulumizza nga bwe baakituwadde. Tekinnakubaganyizibwako birowoozo abakugu abalala. Okusunsula kwa Lancet kukoleddwa ku ekyo eky'Olungereza kyokka nga bwe kyafulumizibwa era kye kirina okujulizibwako ku kiwandiiko kino.

Supplement to: Nassuuna J, Zirimenya L, Nkurunungi G, et al. The effect of BCG revaccination on the response to unrelated vaccines in urban Ugandan adolescents (POPVAC C): an open-label, randomised controlled trial. *Lancet Glob Health* 2024; **12:** e1849–59.

Obufunze

Ennyanjula

Engeri omubiri gye gwanukulamu mu kulwanyisa endwadde nga kireetebwa eddagala ekkulu erigema ery'enjawulo egenze ekendeera mu bantu ba mufuna mpola bw'ogeraageranya n'abalinawo ku nsimbi, n'okugeraageranya ab'omu byalo kw'abo abali mu bibuga. Okugema ne BCG kwongera ku maanyi g'okulwanyisa obulwadde ku maddagala agamu agagema agateerinako kakwate mu bantu abalinawo ku nsimbi. Twakola okuteebereza nti okuddamu okugemwa n'eddagala lya BCG kuyinza okwongera amaanyi mu ngeri omubiri gye gweyisamu ku ddagala erigema eriteerinako kakwate mu baana banna Uganda abasoma.

Enkola

Twakola okugezesebwa okuggule, okufugibwa mu ngeri ey'ekifuulannenge okugeraageranya ebiva mu kugema BCG okugerageranya ne ku butaddamu kugema BCG ku buziyiza bw'okugema okuddirira okutali kukwatagana mu bavubuka ab'emyaka 13–17 abeetabye mu kunoonyereza okw'ekibinja ky'abaana abazaalibwa mu bibuga mu Uganda, nga mu kunoonyereza kuno okugema BCG yawandiikibwa nga yakazaalibwa. Abeetabi baggyibwamu singa baali bafunye eddagala lyonna erigema ery'okugezesa oba ebikwatagana nabyo nga balina emyaka 5 n'okudda waggulu. Enkola ya kompyuta eya 1:1 randomisation yassibwa mu nkola mu REDCap. Abeetabye mu kugezesebwa baggyibwamu singa baali bawandiisiddwa mu kiseera kye kimu mu kugezesebwa okulala; yalina ebyafaayo ebikulu mu bujjanjabi eby'obutabeera na busimu obuziyiza endwadde, oba embeera z'eby'omutwe ez'amaanyi oba endwadde ez'amaanyi ez'ekigero oba ez'amaanyi; baali bamira eddagala erikendeeza abasirikale b'omubiri; nga balina alergy ku biri mu dagala erigema, omuze gw'okufuna enkovu za keloid; nga balina akawuka ka siriimu oba bali lubuto; baali bakyala abeetabye mu kunonyereza kuno abaali bayonsa; oba bwe baba nga baali bategese okukozesa eddagala erinoonyereza, eddagala erigema, ebiva mu musaayi, oba byonna ebigattibwa. Abeetabi abateekebwa mu kibinja ky'okuddamu okugemwa BCG baafuna eddagala lya BCG (Serum Institute of India, Pune, India; 0·1 mL mu lususu, omukono ogwa waggulu ogwa ddyo) mu wiiki 0. Bonna abeetabye mu kugema baafuna eddagala ly'omusujja gw'enkaka (YF-17D; Sanofi Pasteur, okuva e Lyon, Bufalansa eriweebwa nga 0·5 mL mu binywa, mu

mukono ogwa kkono ogwa waggulu), eddagala erigema typhoid eriweebwa mu kamwa (Ty21a; PaxVax, London, UK; kapuso emu buli lunaku amira okumala ennaku ssatu ezikyukakyuka), n'eddagala eriweweeza ku kawuka ka human papillomavirus (HPV) (Merck , Rahway, NJ, USA 0·5 mL mu binywa, ku mukono ogwa kkono ogwa waggulu) mu wiiki 4; n'okugema tetanus ne diphtheria (Serum Institute of India; 0·5 mL, mu binywa, mu mukono ogwa kkono ogwa waggulu) ne HPV booster dose mu wiiki 28. Okugema okulala okwa HPV mu wiiki 8 kwaweebwa abawala abeetabye mu kugema abasukka mu myaka 14 abaali tebannafuna ddagala erigema HPV. Ebikulu ebivuddemu byali nti omusujja gw'enkaka gwanukula ku basirikale b'omubiri ku wiiki 4 oluvannyuma lw'okugema YF-17D, obungi bwa Salmonella enterica serovar Typhi (okuva kati S Typhi) O-lipopolysaccharide (O:LPS)-specific IgG ku wiiki 4 oluvannyuma lw'okugema Ty21a, n'obungi bwa HPV-16 ne HPV-18 L1 protein-specific IgG ku wiiki 4 oluvannyuma lw'okugema HPV. Okukebera ebivaamu ebisookerwako kwakolebwa mu wiiki 8, ne mu wiiki 52 ku tetanus–diphtheria. Okugezesebwa kuno kwawandiisibwa mu ISRCTN Registry (ISRCTN10482904) era kuwedde.

Ebyavaamu

Wakati w'ogwomunaana nga 31 mu 2020 n'ogwekkumi nga 12 mu 2020, twekebejja abasobola okwetabamu 376. Twayingiza abeetabi 300 netubateeka mu bibinja Bibiri; 151 baatekebwa mu kuddamu okugemebwa BCG ate 149 [50%] mu kibinja kyobutaddamu kufuna BCG. 178(59%) ku beetabi 300 baali balenzi ate nga 12(41%) baali bawala. 142(91%) ku beetabi 151 abekibinja kya BCG ne 139(93%) ku beetabi 149 ab'ekibinja ky'obutafuna BCG baamalayo okwekenneenyezebwa mu musomo. Tewali kyatuukawo ku ky'okuddamu okugemebwa okwa BCG, bw'ogeraageranya ku ky'okuddamu okugemebwa awatali BCG, ku byavaayo ebyekenneenyezebwa ku ddagala ly'okugema lyonna. Abaserikale b'omubiri okugema omusujja gw'enkaka (PRNT50) kwalina GMR ya 0·95 (95% CI 0·75–1·19; p=0·62) ate PRNT90 yalina GMR ya 0·94 (0·74–1·19 nga p=0·60); okuddamu kw'abaserikale b'omubiri eri S Typhi O:LPS kwali 0·99 (0·80–1·23; p=0·94); abaserikale b'omubiri okuddamu HPV-16 kwali 0·97 (0·69–1·35; p=0·85) ate ku HPV-18 kwali 1·03 (0·76–1·40; p=0·83); era abaserikale b'omubiri okuddamu ku tetanus kwali 1·13 (0·87–1·47; p=0·36) era kwali 1·00 (0·87–1·16; p=0·97) ku bulwadde bwa diphtheria. Tewaaliwo buzibu bwa maanyi bwonna mu kibinja kyonna.

Entaputa

Tetwafuna bujulizi bwonna bulaga nti okuddamu okugemebwa ne BCG nkola nnungamu okwongera ku maanyi g'omubiri okusisimuka mu kulwanyisa obuwuka obuleeta obulwadde ku ddagala eddala erigema mu mbeera eno.

Abataddemu ensimbi

UK Medical Research Council (MR/R02118X/1)
